# COVID-19 Pandemic Preparedness in Egypt's Teaching Hospitals: A Needs Assessment Study

**DOI:** 10.3389/fpubh.2021.748666

**Published:** 2022-01-17

**Authors:** Muhammad Mostafa Abd El Ghaffar, Marwa Rashad Salem, Mohamed Fawzy Al Soda, Madiha Said Abd El Razik, MarwAli Hassab Tahoon, Mohamed Fathy Tahoon, Basem Eysa, Abd Elfattah Elsayed Hegazy, Abdelkarem Emam Eleraky, Ayman A. Eltayar, Wael Mahmoud Hossam El Din Eldarandly, Dalia Omran

**Affiliations:** ^1^Department of Gastroenterology and Infectious Diseases, Ahmed Maher Teaching Hospital, Cairo, Egypt; ^2^Department of Public Health and Community Medicine, Public Health and Community Medicine, Faculty of Medicine, Cairo University, Cairo, Egypt; ^3^General Organization for Teaching Hospitals and Institutes, Cairo, Egypt; ^4^National Liver Institute, Menoufia University, Shebeen El-Kom, Egypt; ^5^General Surgery Department, Shebin Elkom Teaching Hospital, Shebin El-Kom, Egypt; ^6^Gastroenterology Department, National Hepatology and Tropical Medicine Research Institute, Cairo, Egypt; ^7^General Surgery Department, Elsahel Teaching Hospital, Cairo, Egypt; ^8^Orthopedic Surgery Department, Al Ahrar Teaching Hospital, Zagazig, Egypt; ^9^Intensive Care Department, Damanhour Teaching Hospital, Damanhour, Egypt; ^10^Anaesthesia Department, Matarya Teaching Hospital, Cairo, Egypt; ^11^Faculty of Medicine, Endemic Medicine Department, Cairo University, Cairo, Egypt

**Keywords:** COVID-19, ICU, Egypt, service statistics, teaching hospitals

## Abstract

**Introduction:**

Regular collection and monitoring of data describing the dynamics of the utilization of healthcare services, especially in teaching hospitals (TH), which provide model quality medical services, are critical for COVID-19 pandemic preparedness.

**Methods:**

The researchers analyzed data and information derived from service statistics reports from June 1st to July 15th, 2020 in terms of hospital resources, as well as utilization patterns of beds, ICU, and ventilators, for 11 screening hospitals affiliated with the General Organization of Teaching Hospitals and institutes in Egypt assigned by the Ministry of Health and Population to provide medical care for COVID-19 patients. Hospital indicators in terms of COVID-19 screening services, as well as utilization patterns of inpatient beds, ICU beds, and ventilators were computed.

**Results:**

A total of 78,869 non-medical personnel and 2,176 medical personnel were presented with COVID-19 triage symptoms. Investigations conducted in the targeted 11 hospitals delineated that 22.2% of non-medical personnel and 27.9% of medical personnel were COVID-19 PCR-confirmed cases. The inpatient bed occupancy rate was 70% for non-medical patients and 67% for medical staff patients. For ICU, the bed occupancy rate was 92 % for non-medical patients and 88% for medical patients. Among the confirmed cases, 38% of medical patients utilized a ventilator vs. 36% of medical personnel cases. Hospital ranking according to utilization pattern among non-medical personnel, Hospital H ranked first in terms of the high load of screening services. Hospital C ranked first regarding the number of confirmed cases, whereas Hospital D ranked first for high ICU utilization among all teaching hospital ICU cases. With respect to medical personnel, Hospital G ranked first for the high load of screening services for the total studied cases. Hospital G ranked first for the number of confirmed cases. Hospital B ranked first regarding high ICU utilization among all teaching hospital ICU cases.

**Conclusion:**

Teaching hospitals have demonstrated preparedness for the COVID-19 pandemic by maintaining an inpatient bed occupancy rate of 70% or less and ventilator utilization at <40% of confirmed cases. However, the ICU bed occupancy rate was more than 90% indicating a shortage of resources. In addition, there is variance across hospitals regarding caseload for resource reallocation decisions.

## Introduction

As the world responds to the emerging COVID-19 pandemic, the change in current global public health priorities is revealing crucial weaknesses in global health systems (1). Worldwide, there are growing concerns about the need for hospital beds that will overwhelm national capacity, placing severe strains on the health care system and limiting access to essential care (2). A decrease in the number of hospital beds, intensive care beds, and ventilators representing weaknesses in healthcare systems worldwide has been reported (3, 4). Critical care capacity in low- and middle-income countries (LMICs) was inadequate prior to the pandemic, and they are at risk of not being able to cope with the estimated rise in critically ill COVID-19 patients, with current estimates of 0.1–2.5 ICU beds per 100,000 people are open (3). However, shortages have spread to even more resourced health systems around the world (5). Since healthcare systems (mainly hospitals) are the first line of defense to face this pandemic, the COVID-19 negative effects imposed exceptional challenges to these systems and posed a direct threat to their workers (6), especially in developing countries like Egypt, which suffered from a lack of resources and a weak health system prior to the pandemic (7).

The Egyptian government abandoned individual hospitals in every governorate to be assigned as quarantine hospitals for COVID-19 patients (Isolation Hospitals) after the first outbreak of COVID-19 in Egypt on February 14 (8), but infections continued to rise as the number of confirmed cases in Egypt increased. These statistics put additional pressure on the already overburdened public health sector, which has already received the majority of the cases and is overstretched (9).

As a result, regular collection and review of various data, such as healthcare capacity and usage, is critical for informing COVID-19 pandemic preparedness and response and organizational decision-making on service delivery (10). In the current study, the researchers attempted to investigate the situation in some of the COVID-19 screening hospitals in terms of inpatient beds, ICU beds, and ventilator utilization rates. Traditionally, the previously mentioned indicators were to provide policymakers with feedback on the COVID-19 response and to be used by decision-makers. We documented the number of hospitalized patients, either medical or non-medical personnel, with COVID-19 as a vital pulse on the severity of the disease in our community.

## Materials and Methods

A statistical report was written utilizing service statistics from 11 screening hospitals of the General Organization of Teaching Hospitals and Institutes (GOTHI) in Egypt. The Ministry of Health and Population (MOHP) assigned the screening hospitals of GOTHI to provide medical care for patients with COVID-19 infection in Egypt. All adult patients (≥18 years), including health care providers, who attended the studied Teaching Hospitals and Institutes with COVID-triage symptoms during the period from June 1 to July 15, 2020, whether or not they were admitted to the (GOTHI), were included in the current study. This range of dates falls within the first wave of the pandemic. We extracted data, including the numbers of confirmed cases, discharges, new deaths, and severe cases, during the study duration. There is no sampling at all, as all Eleven teaching hospitals in Egypt were included in the study, and all COVID-19 cases were attended to at teaching hospitals during a specific period of time.

Operational definitions of variables, terms, and indicators (11).

*We computed the following indicators according to the following formulas:*

1. Inpatient bed occupancy rate =
  Total number of inpatient days for a given period × 100
  Available beds × Number of days in the period
2. ICU bed occupancy rate =
  Total number of ICU days for a given period × 100
  Available ICU beds × Number of days in the period
3. Ventilator utilization rate = percent of patients with confirmed cases who used the ventilator.
4. In-hospital mortality rate for COVID-19 cases.

Percentage of hospital deaths of confirmed COVID-19 cases after 48 hours of admission during the specified time period to the total number of COVID-19 confirmed cases admitted to the hospital in the same specified time period.

## COVID-19 Case Definitions

The Egyptian Ministry of Health published a comprehensive guide for diagnosing and treating COVID-19. Patients with the COVID-19 infection are divided into mild, moderate, and severe cases (8).

### Mild COVID-19 Cases

Symptomatic case with lymphopenia or leucopenia with no radiological signs for pneumonia.

### Moderate COVID-19 Cases

The patient presents with pneumonia manifestations on radiology associated with symptoms and/or leucopenia or lymphopenia.

### Severe COVID-19 Cases

If any of the following criteria are present:

1. RR > 30
2. SaO_2_ < 92 at room air
3. PaO_2_/FiO_2_ ratio < 300
4. Chest radiology shows more than 50% lesion or progressive lesion within 24–48 h.
5. Critically ill if SaO_2_ < 92, or RR > 30, or PaO_2_/FiO_2_ ratio < 200 despite oxygen therapy (12).

### Statistical Analysis

Pre-coded data were entered and analyzed using Excel 2010 manufactured by Microsoft. Categorical variables were expressed in frequency and percentages. All the teaching hospitals and institutes were coded in letters A, B up to K for easy manipulation of data as displayed in Table A.

**Table A:** Setting Code for each of the studied teaching hospital.

**No**.	**Hospital code**
1	A
2	B
3	C
4	D
5	E
6	F
7	G
8	H
9	I
10	J
11	K

### Ethical Approval

The Ethical Review Committee of the General Organization of Teaching Hospitals and Institutes (GOTHI) in Egypt revised and approved the study protocol.

## Results

Table 1 illustrated these 11 teaching hospitals that provided screening tests for ~78,869 non-medical personnel who attended the hospital with COVID-triage symptoms during the period (June the 1st to July the 15th, 2020). Among the screened suspected non medical cases, 22% were confirmed as clinical cases of COVID-19. Among the confirmed cases, 72.2% were mild and 23.2% were moderate and severe cases. Out of the confirmed cases, 4.6 % were admitted to the ICU of the corresponding hospital.

**Table 1:** The percentage of PCR confirmed COVID-19 cases among total suspected cases presented with COVID-19 symptoms to teaching hospitals (June 1st to July 15th, 2020).

**Type of patients**	**No. of suspected cases presented with COVID triage symptoms**	**No (%) of confirmed cases**	**No (%) Confirmed cases (mild)**	**No (%) Confirmed cases (moderate and sever cases admitted inpatients)**	**No (%) Confirmed cases need ICU**
Cases not including medical staff	78,869	17,508 (22.2%)	12,643 (72.2%)	4,063 (23.2%)	802 (4.6%)
Medical staff members	2,176	609 (27.9%)	418 (68.6%)	174 (28.6%)	17 (2.8%)

The corresponding figures for Medical Staff cases were as follows: 10 teaching hospitals provided screening tests for about 2,176 medical staff who attended the hospital with COVID-triage symptoms during the period (June the 1st to July the 15th, 2020). Among the screened cases, 27.9% were confirmed as clinical cases of COVID-19. Among the confirmed cases, 68% were mild and 28.6% were moderate and severe cases. Among the confirmed cases, 2.8% were admitted to the ICU in the corresponding hospital (Table 1).

Inpatient bed occupancy and ICU bed occupancy and ventilator utilization rates for COVID-19 confirmed non-medical and medical admitted to the teaching hospitals (June 1st to July 15th, 2020) are illustrated in Table 2. The bed occupancy rate for COVID-19 confirmed was 70% for non-medical cases, and it was 92% for ICU admissions. The percent of confirmed cases utilizing ventilators was 38%. Corresponding figures for medical staff member cases were 67% for in-patient cases, 88% for cases admitted to ICU. The percent of confirmed cases that utilized ventilators was 36%.

**Table 2:** Inpatient bed occupancy and ICU bed occupancy and ventilator utilization rates for COVID-19 confirmed cases admitted to teaching hospitals (June 1 to July 15, 2020).

**Type of patients**	**Inpatient bed occupancy rate**	**ICU bed occupancy rate**	**Ventilator utilization rate**
Cases not including medical staff	70%	92%	38%
Medical staff members	67%	88%	36%

Table 3 showed that 20% were reported as discharged cases and deaths after 48 h of admission were 7.3% for the total cases of non medical personnel. Corresponding figures for medical staff member cases were 60.4%, discharged cases 2.9% deaths after 48 h.

**Table 3:** Percent of teaching hospital discharges for COVID-19 confirmed cases and in-hospital mortality rate throughout the period (June 1 to July 15, 2020).

**Type of patients**	**Total number of confirmed cases**	**No (%) discharged cases**	**Total number of deaths**	**In hospital mortality rate**
Cases not including medical staff	17,508	3,454 (20%)	1,290	7.3%
Medical staff	609	368 (60.4%)	18	2.9%

**Figure 1:**
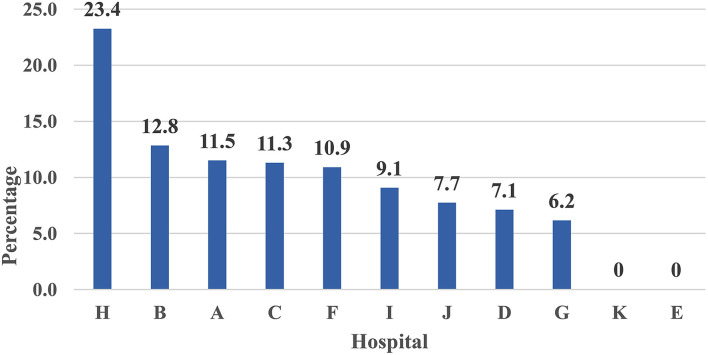
Percent distribution of screened non-medical personnel with COVID-19 triage across 11 Teaching hospitals—Egypt: (June 1 to July 15, 2020)—Rank ordering.

Figure 1 illustrates the caseload per teaching hospital for screening tests for COVID-19 triage non-medical personnel. The percent contribution for a hospital in screening tests services showed the Hospital H ranked first, Hospital B ranked second, Hospital A ranked third, and Hospital E ranked last.

**Figure 2:**
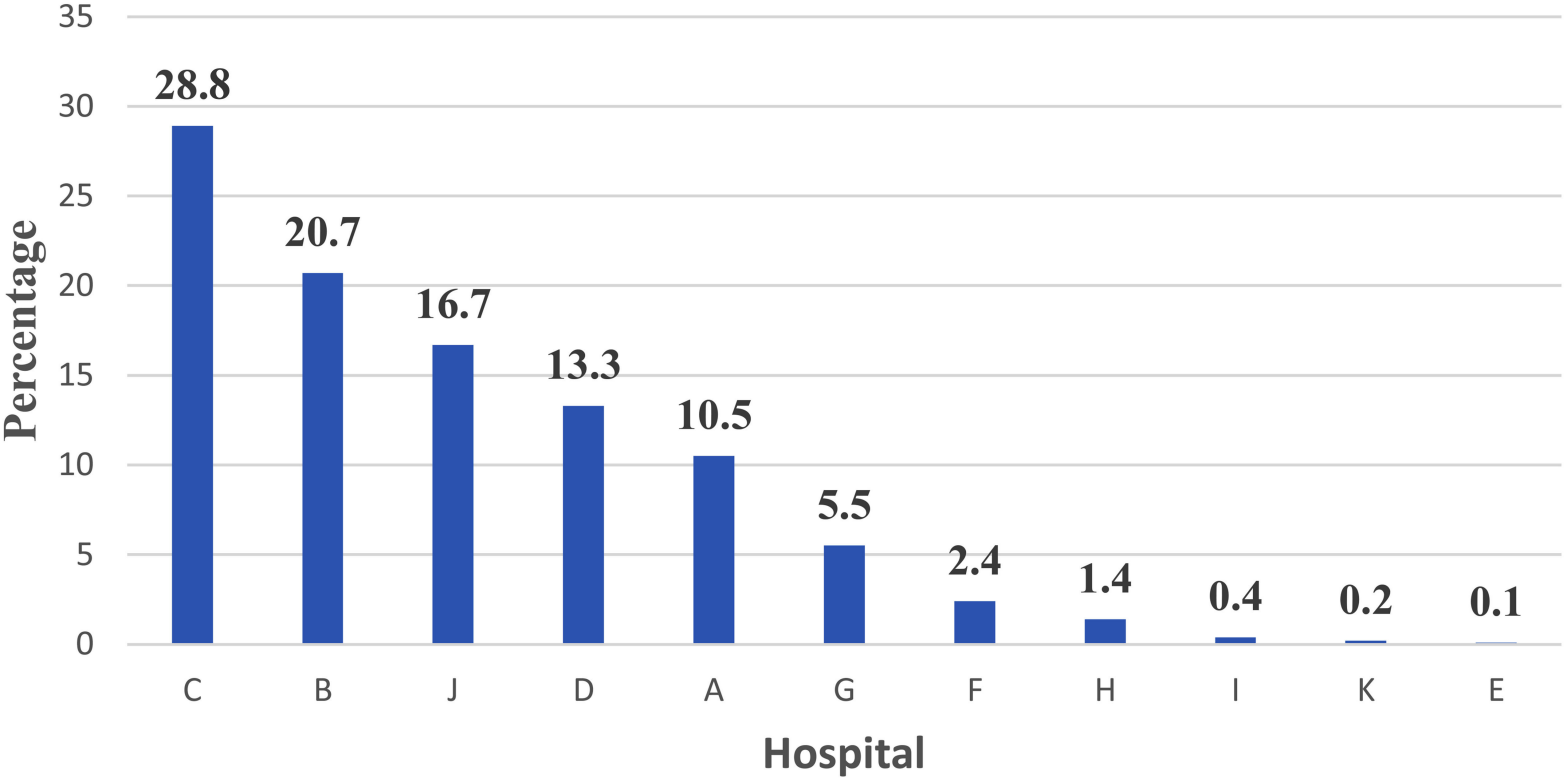
Percent distribution of PCR confirmed COVID-19 across 11 Teaching hospitals among non-medical personnel—Egypt: (June 1 to July 15, 2020) —Rank ordering.

On the contrary, the distribution of PCR confirmed COVID-19 across 11 Teaching hospitals showed the Hospital C ranked first, Hospital B ranked second, and Hospital J ranked third for non-medical personnel as shown in Figure 2.

**Figure 3:**
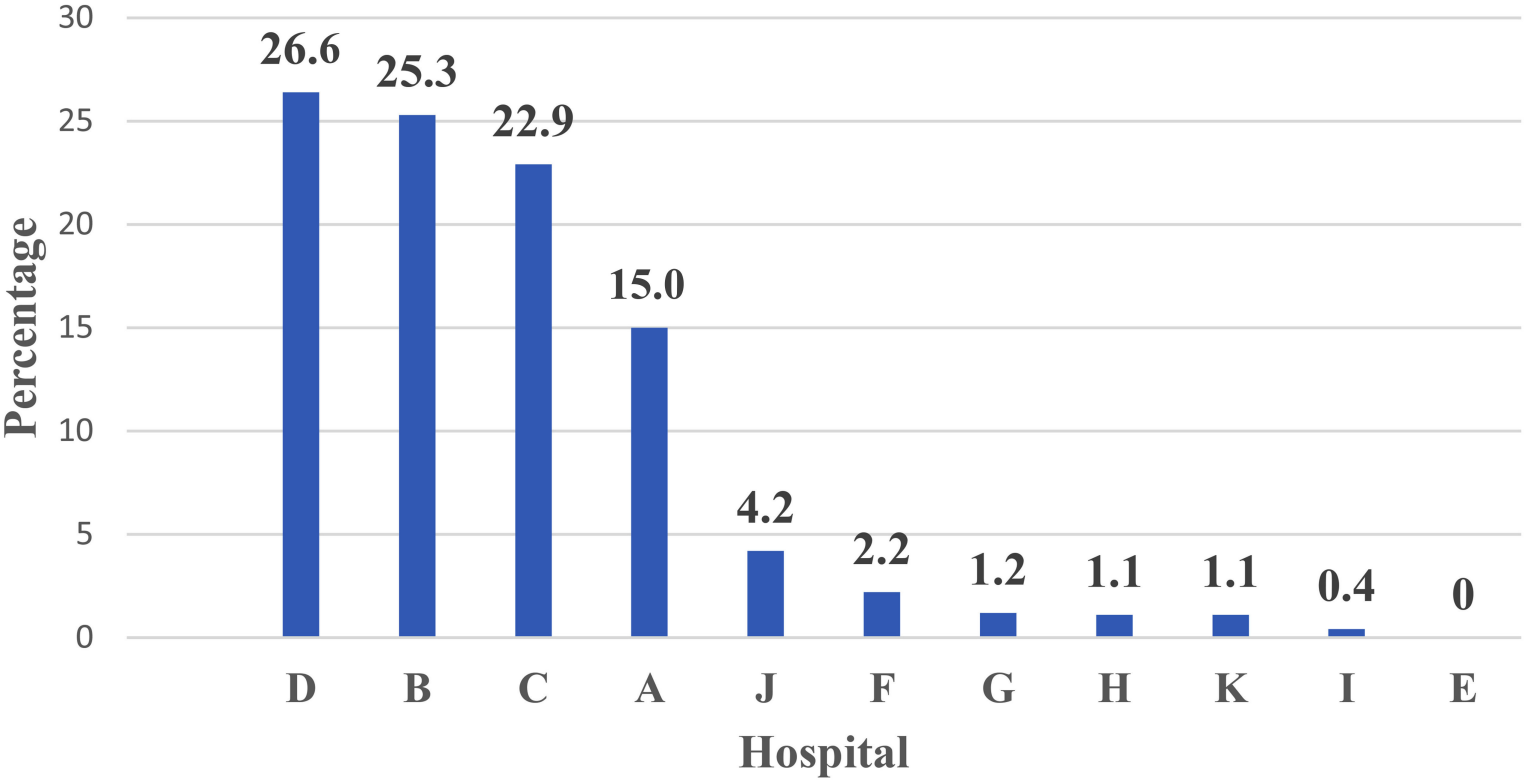
Percent distribution of PCR confirmed COVID-19 across 11 Teaching hospitals among non-medical cases who utilized ICU—Egypt: (June 1 to July 15, 2020) —Rank ordering.

Figure 3 shows the percent distribution of PCR confirmed COVID-19 across 11 Teaching hospitals among non-medical cases who utilized ICU—Egypt: (June 1 to July 15, 2020). Hospital D ranked first, hospital B ranked second and C ranked third.

**Figure 4:**
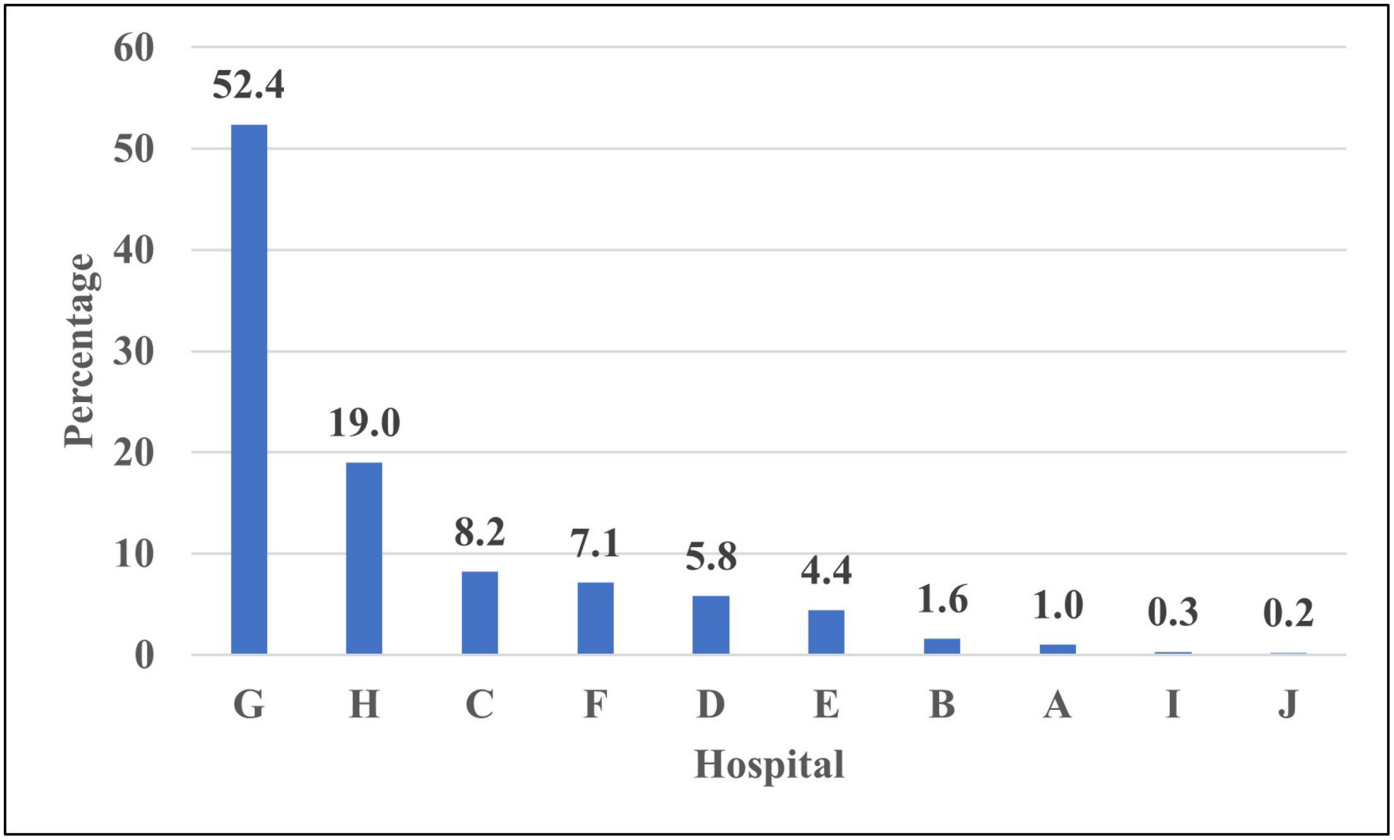
Percent distribution of screened medical personnel with COVID-19 triage across 10 Teaching hospitals—Egypt: (June 1 to July 15, 2020) —Rank ordering.

Figure 4 illustrates the caseload per teaching hospital for screening tests for COVID-19 triage medical cases. The percent contribution for a hospital in screening tests services showed that Hospital G ranked first, Hospital H ranked second, while Hospital C ranked third. Hospital J ranked last.

**Figure 5:**
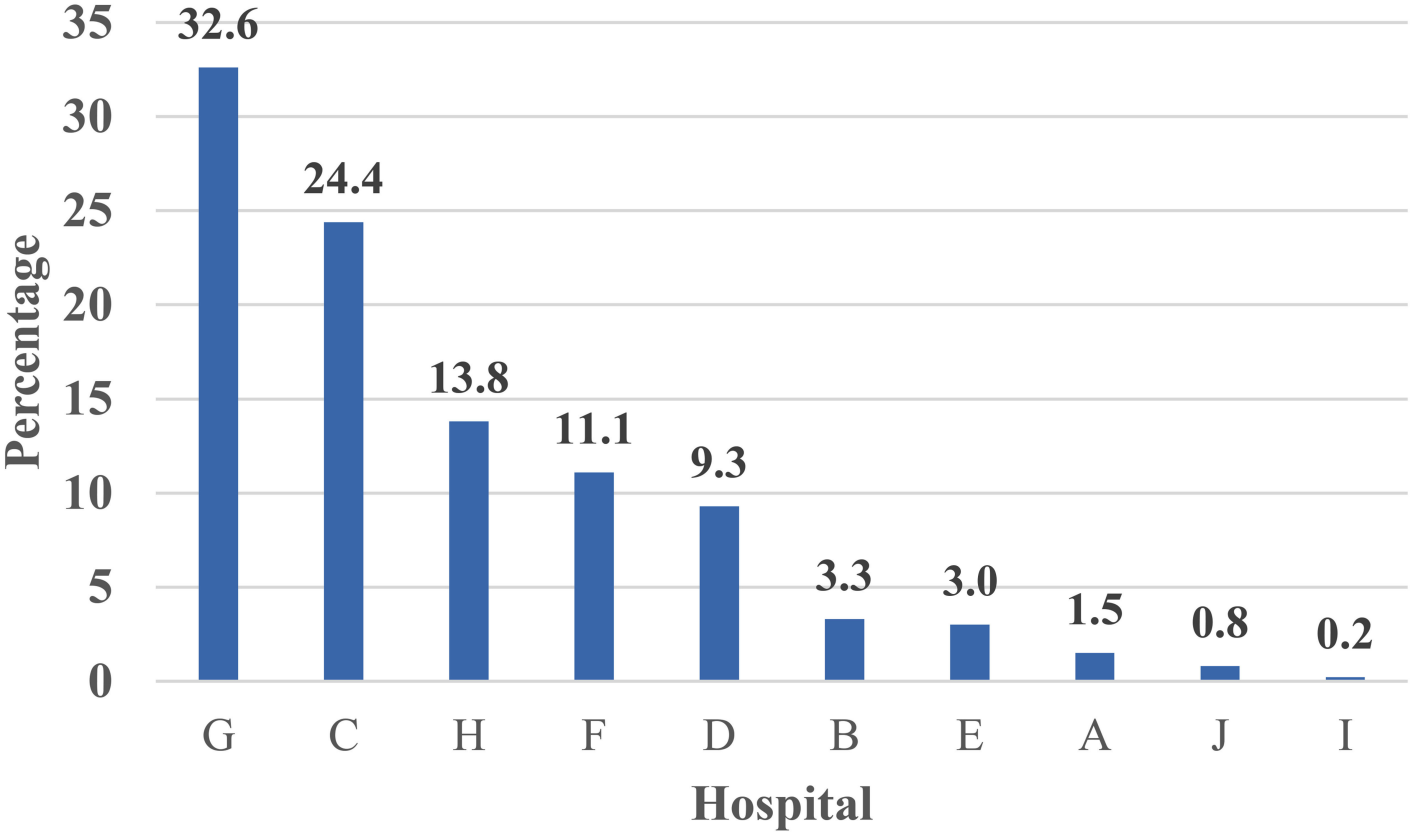
Percent distribution of PCR confirmed COVID-19 across 10 Teaching hospitals among medical personnel—Egypt: (June 1 to July 15, 2020)—Rank ordering.

**Figure 6:**
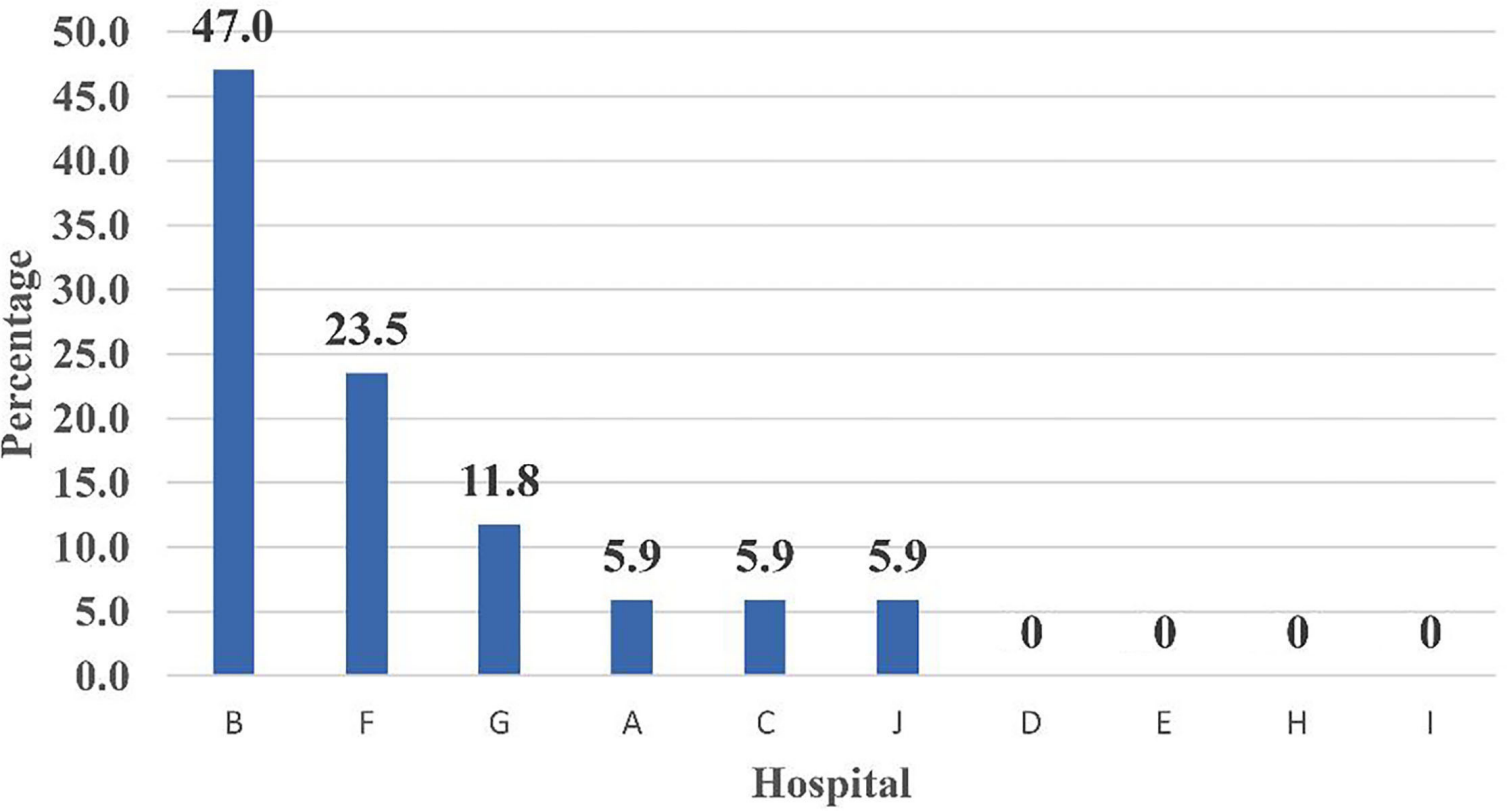
Percent distribution of PCR confirmed COVID-19 across 10 Teaching hospitals among medical cases who utilized ICU—Egypt: (June 1 to July 15, 2020)—Rank ordering.

The percent distribution of PCR confirmed COVID-19 across 10 Teaching hospitals among medical personnel—Egypt is shown in Figure 5. Hospital G reported the highest percentage of confirmed cases, followed by hospital C, while Hospital I ranked last. Figure 6 illustrates the percent distribution of PCR confirmed COVID-19 across 10 Teaching hospitals among medical cases who utilized ICU—Egypt: (June 1 to July 15, 2020). It is clear from the figure that hospital B ranked first followed by hospital F (Figure 6).

## Discussion

The current study presented a model for hospital care dynamics for COVID-19 patients in 11 teaching hospitals in Egypt from June 1 to July 15, 2020. The study focused on the patient cycle from the triage stage of the COVID-19 conduction of screening tests, hospital care, and discharge. Additionally, the study used specific output indicators to present utilization patterns for hospital resources as in-patient hospital bed occupancy and ICU bed occupancy, and percent of admitted cases utilized ventilators. The outcome indicator used in the study was the percent of discharged cases out of the total admitted hospital cases. The impact indicator used in the study was the in-hospital mortality rate. The indicators used to measure the dynamics of the patient cycle were determined on data derived from 11 hospitals during a specific period of COVID-19-Wave I. The indicators in the article were organized according to patient cycle steps. The caseload per teaching hospital for screening tests for COVID-19 triage for non-medical and medical staff cases could interpret hospital capacity to conduct screening tests. The percent contribution for a hospital in screening tests services showed that Hospital H ranked first, Hospital B ranked second, and Hospital ranked third. With regard to medical staff cases, Hospital G ranked first, Hospital H ranked second, and Hospital C ranked third. These hospitals have priority in providing resources for COVID-19 screening services to deal with the high caseload targeting screening.

In contrast, for medical staff cases; Hospital G has priority for providing in-patient (hospital beds) services resources to maintain a low bed occupancy to respond to the high flow of confirmed cases.

The inpatient bed occupancy rate for COVID-19 confirmed cases has reached 70% for non-medical cases. The corresponding figure for medical staff cases was 67%. ICU bed occupancy rate for patients was more than 90%, which is an alarming signal of a shortage of resources. This was corresponding to the figure reported by the Minister of Health and Population in Egypt (84%) by COVID-19 patients (13). In the current study, variance across hospitals regarding ICU occupancy rate guides for resource reallocation decisions where a recent study conducted by Labib et al. (14) in Egypt to assess ICU preparedness during the COVID-19 pandemic revealed that the overall preparedness in both pediatric and adult ICUs was 54%. This situation could be due to the current belief that COVID-19 mainly affects adults with more severe cases than those observed among children.

Impact indicators and cases of COVID-19 in-hospital mortality among non-medical and medical staff cases indirectly reflect multiple independent variables as hospital services and characteristics of the patients as age, sex, and comorbidities.

The distribution and severity of COVID-19 are substantially dissimilar in different parts of the world. The in-hospital mortality rate of cases admitted to the ICU is between 23.4 and 33%. The mortality rate among patients receiving mechanical ventilation is 43 to 67% and is close to 70% among patients older than 60 years (15). In the current statistical report, from 17,508 patients without medical staff, 1,290 cases died (7.3%). An analysis of American Hospital Association data with COVID-19 data revealed an association between low hospital resources and mortality (16). On the contrary, given their daily interaction with infected patients, medical staffs are at risk of contracting COVID-19. The average incidence of COVID-19 infection among healthcare staff has been about 10%. In the United States, this percentage was 18%, while in China it was 4% and 9% in Italy (17). The availability of high-quality personal protective equipment (PPE) kits, proper use of PPE, and infection prevention training programs are all important factors in lowering infection rates. As revealed from the current study, a higher percentage of medical staff 27.9% were confirmed as clinical cases of COVID-19. This high percentage came according to the Egyptian government official reports, as the number of infected and deceased Egyptian doctors as well as allied health workers increased compared to the general population. On the frontlines of protection against the COVID-19 pandemic protection, health care workers are exposed to not only COVID-19 infection due to their daily contact with infected individuals, but also psychological distress, long work hours, exhaustion, workplace stigma, and physical abuse. Policymakers in Egypt are acquainted with the high risk of exposure of the medical teams to COVID-19 infection. They adopted different strategies to reduce the risk of exposure of medical staff to COVID-19 infection. The Minister of Health and Population in Egypt has allocated a floor in each isolation hospital with a capacity of 20 beds, to treat infected medical staff, as part of the Ministry's efforts to protect its medical teams to confront them with the emerging Coronavirus, providing psychological, and administrative support. Additionally, infection control teams in hospitals are working daily to review the stock of preventive supplies, and to ensure that medical teams are following precautions to prevent any medical staff from being infected with the virus. The Egyptian Minister demonstrated that they distributed large quantities of preventive supplies to hospitals, besides conducting weekly webinars targeting all health care workers to keep them updated with COVID-19 management protocols and infection control measures (18). Despite the efforts of MOHP, the current study revealed high hospital mortality among medical staff. This needs further investigations into the reason behind the high in-hospital mortality rate among HCWs in Egypt.

The study focused on information derived from 11 teaching hospitals. Hospitals' presentation and contribution to providing specific services to COVID-19 cases were crucial for policy and decision-makers. The indicators indirectly reflect the demand side for teaching hospitals. The demand side is measured by the percent contribution of each hospital in providing screening tests to COVID-19 triage cases. This indicator delineated caseload at an outpatient level and was influenced by the catchment area of each hospital, and the level of acceptability of specific hospital services by the served community. The availability and quality of services contribute to the magnitude of such indicators. The supply side is related to the availability of qualified medical health human resources, screening Lab services, ICU, and ventilators. The interaction between demand and supply-side was measured by outcome and impact indicators as discharged cases and in-hospital mortality. To avoid overload and saturation, one of the primary problems was to manage health resources quickly and efficiently. This is especially relevant in nations where there was a dearth of accessible beds (which filled quickly in the early days of epidemics), as well as a shortage of health professionals (who were overworked) (19, 20).

## Conclusion

Teaching hospitals have demonstrated preparedness for the COVID-19 pandemic by keeping the bed occupancy rate at 70% or less and ventilator utilization at <40% of confirmed cases. However, the bed occupancy rate in ICU was more than 90% indicating a shortage of resources for critical clinical cases of COVID-19 cases. Impact indicators as in-hospital mortality among non-medical and medical staff cases indirectly reflect multiple independent variables as hospital services and patient characteristics such as age, sex, and comorbidities.

## Clinical Implications

In clinical areas, the findings of this study can help hospital administrators and hospital leaders to identify the strengths and weaknesses of hospital preparedness for suspected and confirmed cases of COVID-19. The hospital can be guided in designing a continuing education program that would enhance hospital preparedness. Identifying the strained aspects of hospital preparedness is crucial to improving and strengthening their work in the prevention, control, management, and containment of the COVID-19 pandemic.

## Recommendations

- Hospitals that have priority in providing lab resources for screening tests were Hospitals H, B, and G. Facilities are needed to manage clinically confirmed cases of Hospitals G, C, and H.
- Hospitals that have priority in providing in-patient (hospital beds) services resources for maintaining low bed occupancy to respond to a high flow of confirmed cases were Hospitals C, B, and J.
- Hospitals that have priority in providing in-patient (ICU) services resources for keeping response to critical cases were hospital D followed by hospital B and C.

## Strengths

- The study was conducted during the pandemic and concerned with the supply side of hospitals to control comorbidities and deaths.
- The information included both medical and non-medical groups regarding diagnostic tests, ICU utilization, and the use of ventilators.
- The study could measure the preparedness of hospitals during the pandemic, which is an important issue to be considered in health care.
- It is a policy-oriented research as it has its implications for policymakers at hospitals and MOHP-Headquarter.
- The study included different departments of the Ministry of Health: surveillance of COVID-19, curative care sector, logistic managements (keeping a good number of ventilators), and pharmaceutical sector to supply medications according to the flow of cases.
- The study has specific implications for the medical syndicate, as the study displayed information about medical staff.
- The study demonstrated different indicators to be used for monitoring and evaluating the performance of hospitals during pandemics.
- Future studies in the hospitals may be conducted to monitor the changes over time.

## Limitations of the Study

The current study findings should be viewed with respect to the following limitations. First, the data were derived from the hospitals during the pandemic. Therefore, there are a limited number of variables that may contribute to the analysis and the development of more indicators. No information is available for the characteristics of patients such as age, sex, comorbidity to justify the use of hospital beds, ICU, and ventilators as well as in-hospital mortality. Further assessment during different phases of the COVID-19 pandemic is required. Second, the results cannot be generalized as it is not an epidemiological study, since it is operation research, health services management-oriented study only the methodology can be generalized and to be replicated in other medical settings.

## Data Availability Statement

The original contributions presented in the study are included in the article/supplementary material, further inquiries can be directed to the corresponding author/s.

## Ethics Statement

The studies involving human participants were reviewed and approved by Teaching Hospitals-Egypt. Written informed consent for participation was not required for this study in accordance with the national legislation and the institutional requirements.

## Author Contributions

MuA conceived the study, contributed to managing the literature searches, and data management. MS assisted with the literature search and writing. MaA contributed to data analysis and results writing. DO and MoA contributed to data collection and writing. MaT, MoT, BE, AH, AbE, WE, and AyE shared in data collection, drafting, and approving the final manuscript in the study. All authors contributed to the article and approved the submitted version.

## Conflict of Interest

The authors declare that the research was conducted in the absence of any commercial or financial relationships that could be construed as a potential conflict of interest.

## Publisher's Note

All claims expressed in this article are solely those of the authors and do not necessarily represent those of their affiliated organizations, or those of the publisher, the editors and the reviewers. Any product that may be evaluated in this article, or claim that may be made by its manufacturer, is not guaranteed or endorsed by the publisher.
